# Guiding Ebola patients to suitable health facilities: an SMS-based approach

**DOI:** 10.12688/f1000research.6105.1

**Published:** 2015-02-12

**Authors:** Mohamad-Ali Trad, Raja Jurdak, Rajib Rana

**Affiliations:** 1 Medecins Sans Frontieres, Paris, France; 2 Department of Infectious Diseases, Monash Medical Centre, Melbourne, Australia; 3 Commonwealth Scientific Industrial and Research Organisation, Brisbane, Australia; 4 University of Southern Queensland, Brisbane, Australia

**Keywords:** ebola, SMS, text message, health service

## Abstract

Access to appropriate health services is a fundamental problem in developing countries, where patients do not have access to information and to the nearest health service facility. We propose building a recommendation system based on simple SMS text messaging to help Ebola patients readily find the closest health service with available and appropriate resources. The system will map people’s reported symptoms to likely Ebola case definitions and suitable health service locations. In addition to providing a valuable individual service to people with curable diseases, the proposed system will also predict population-level disease spread risk for infectious diseases using crowd-sourced symptoms from the population. Health workers will be able to better plan and anticipate responses to the current Ebola outbreak in West Africa. Patients will have improved access to appropriate health care. This system could also be applied in other resource poor or rich settings.

## Background

The current Ebola virus outbreak in West Africa that originated in Guinea in December 2013, and now has spread to two neighbouring countries, is still uncontained and remains the largest known Ebola virus disease outbreak to date. Some of the challenges identified include the geographical spread of cases, people’s movement between affected countries, the transport and burial of bodies by relatives, the weak healthcare systems, severe human resource constraints, lack of proven therapeutics, and lack of community engagement and cooperation ^1, 2^.

There have been numerous reports where probable Ebola-infected patients had to be driven away from health care facilities due to lack of bed availability. Furthermore, mobility of a positive case imposes further public health risks ^1^. The current Centre for Disease Control and Prevention (CDC) recommendations to stop the spread of Ebola includes: case finding and diagnosis, case isolation and contact tracing, and safe burial practices ( http://www.cdc.gov/vhf/ebola/resources/index.html). Difficult access due to remoteness and lack of transportation ^3^ further widen the gap between the patient and the healthcare provider. Access to information that guides patients to the nearest facility with appropriate resources is another challenge. There is currently no known system that can improve this access.

We propose building a recommendation system based on simple SMS text messaging to help Ebola patients readily find the closest health service with available and appropriate resources. The system will map people’s reported symptoms to likely Ebola case definitions and suitable health service locations. In addition to providing a valuable individual service to people with curable diseases, the proposed system will also predict population-level disease spread risk for infectious diseases automatically using the crowd-sourced symptoms from the population.

**Figure 1.**
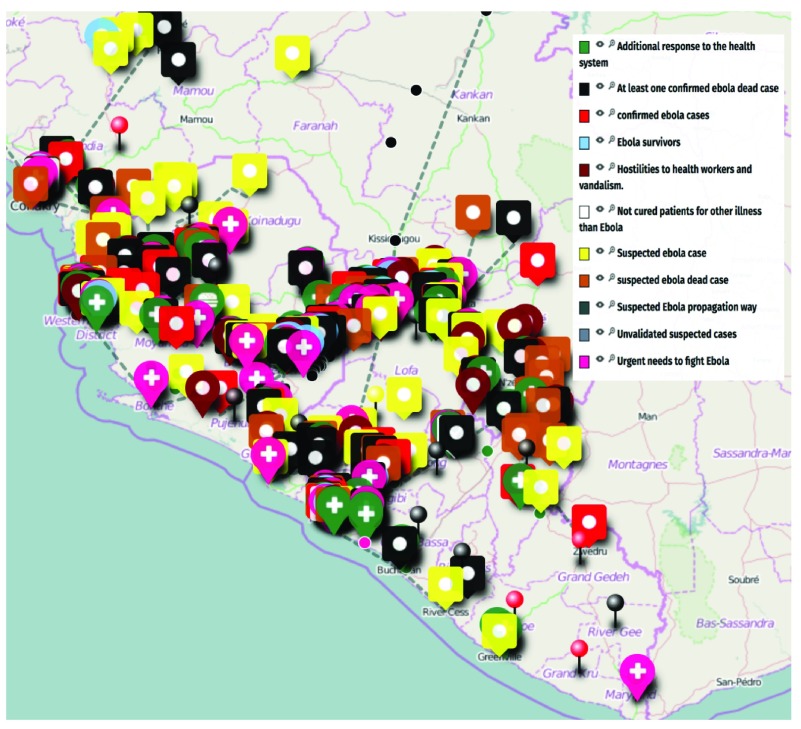
Ebola tracking in Sierra Leone, Liberia and Guinea by Cédric Moro ( http://umap.openstreetmap.fr/en/user/MORO_CEDRIC/).

This will be extremely valuable for health workers to better plan and anticipate responses to the current Ebola Outbreak in West Africa and hopefully prevent many new cases. An Ebola tracking service has been developed by Cedric Moro (See Figure 1), who has been collecting data using the Whatsapp platform to produce maps showing the spread of infection. The key limitations of using Cedric Moro’s approach is that Whatsapp requires an advanced Smartphone, so it can capture a biased sample of the population only. In addition, it only tracks Ebola and does not predict the spread trajectory.

In contrast, simpler and more affordable mobile phones are highly utilized in affected West African countries. For example, in Guinea mobile phone penetration is as high as 71 percent ( http://www.budde.com.au/Research/Guinea-Telecoms-Mobile-and-Broadband-Market-Insights-and-Statistics.html). Therefore, mobile phones can potentially be an inexpensive yet effective way of communication in overcrowded urban or remote settings. For example, the Somali experience with this technology has been encouraging in controlling cholera outbreaks. ( http://blogs.oxfam.org/en/blogs/12-11-13-mobile-phones-help-tackle-cholera-somalia).

## General approach

**Figure 2.**
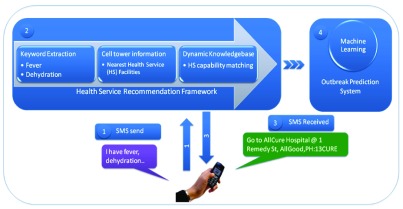
Recommendation Framework.

The workflow of the proposed end-to-end system is shown in Figure 2. The use case for the patients will be straightforward. Using any basic mobile phone they can send a text message about their symptoms based on CDC case definition ( http://www.cdc.gov/vhf/ebola/resources/index.html) to a designated toll-free number. The message once received will be analysed with Natural Language Processing Algorithms to extract the symptoms. Patient location will be approximated by the available cell-tower information or provided post-code or village name. Symptoms will then be fed to the classification module to determine whether it is a suspected Ebola case. The classification module maps the reported symptoms to the CDC provided clinical symptoms of Ebola. If suspected, health service(s) within a chosen radius (configurable) with sufficient resources will be recommended using a return text message. This message will contain name, location and phone number of the health service(s).

The symptoms, which occur in Ebola, are very similar to those, which occur in many other diseases in tropical Africa ( https://web.stanford.edu/group/virus/filo/humandiseases.html). Therefore, our classification module will take into account available meta information, such as the spatial extent of the current outbreak while determining the suspected case. If the symptoms only partially matches the case definition of Ebola, but the patient is based in an Ebola affected zone, he or she will be recommended to visit the health service for full check-up. The patients would then be texted a few hours later asking whether they went to the recommended hospital and if so to provide feedback.

To obtain information about currently available facilities in a health service, we will adopt a phased approach. The knowledge base will be initially constructed using the location and contact number of the health services extracted from web portals using an automated web crawler; later on, we will use both positive and negative patient feedback to infer the current facilities at the service. For example, if patient feedback shows the recommendation was useful, we will assume that the health service has capacity to admit more patients. Otherwise, we will assume that the health service is out of capacity and keep the status for some time (which would need to be determined). However, there will be a provision for the health services to update their information anytime regarding resource, location (e.g. NGO field clinics) in the knowledge base using text messages. At the server end the text messages indicating information update will undergo Natural Language Processing algorithms to extract the key words (e.g., how many beds available) and the knowledge base will be updated accordingly. Once the information is updated, the health services/patients will be notified using a reply text message.

The text messages received from the population of end-users will be continuously analysed at the back-end to predict the outbreak risk into the future and its spread pathways. The locations of users extracted from the text messages will be used to estimate their orbits of movement. These locations will be coupled with the inferred patient conditions to predict the geographic extent of disease-spread risk.

## Discussion

### Need for a communication officer

We use inference (from positive or negative feedback) to predict the capacity of a health service to accommodate more patients. Such a method could be useful if the service cannot update their service status. In order to significantly improve the performance of our system we would recommend appointing a communication officer in every service, who will regularly update the service (number of beds, doctors, medicine etc.) availability in the system. Update interval can be hourly or even 4-hourly - a time unit that would be variable and dependent on the facility’s capacity.

### When recommendation is not possible

In some cases the system may not be able to recommend health services within the nominated radius. In those unsuccessful cases the system will store the patient information and as soon as any availability is spotted, a follow-up text message will be sent to the patient.

### Recommendation across border

When Ebola outbreaks take place simultaneously across bordering countries, such as in Liberia and Sierra Leone, our recommendation system can be adapted to recommend health services across borders, which could potentially help managing patient load.

## Conclusion

The current Ebola outbreak is shaping up to be a large-scale problem, not only in the three affected West African countries, but also with cases starting to appear in Western countries such as Spain and the USA. The CDC recommendations for controlling the spread further are at the source, i.e. in West Africa. Given the large spatial scale of the disease, any assistive technology should be equally prevalent and widely used in the target countries. It is particularly for this reason that we believe and advocate for the use of simple SMS messaging as a mechanism to guide Ebola patients to suitable health facilities.

## Project stage

Currently we are actively seeking funding to commence the project. We are approaching the Gates Foundation in addition to mobile phone operators in West Africa. We are also looking for international and local partner organisations operating in the affected areas.
