# Supplementary material for: Guiding Ebola patients to suitable health facilities: an SMS-based approach
Source: F1000Res. 2015 Feb 12;4:43. [Version 1] doi: 10.12688/f1000research.6105.1 (PMC4358411; doi:10.12688/f1000research.6105.1)
Supplement: Peer review history

# Peer review: Referee response for version 1

**DOI:** 10.5256/f1000research.6537.r7655

**Published:** 2015-03-10

**Authors:** Patrick Charles

**Affiliations:** 1 Department of Infectious Diseases, Austin Health, Melbourne, Vic, Australia

The manuscript describes a proposed SMS-based system to use in locations where Ebola virus disease (EVD) is occurring to help direct possible cases to the nearest treatment centre that has beds available at the time.

This is an interesting and useful idea and would work well in Africa where mobile phone technology is widely available.

The manuscript is well written and the idea is presented well.

I have read this submission. I believe that I have an appropriate level of expertise to confirm that it is of an acceptable scientific standard.

# Peer review: Referee response for version 1

**DOI:** 10.5256/f1000research.6537.r7657

**Published:** 2015-02-26

**Authors:** Corinna Schmitt

**Affiliations:** 1 Department of Informatics, University of Zurich, Zurich, Switzerland

The authors present a good idea how toestablish a SMS-based approach to guide Ebola patients to health facilities. The idea is great and the strategy sound reasonable, but the following issues must be clarified in more detail:

- How do the authors guarantee that the patient will get the information of the most nearest facility?
- What is with the support of poor regions with low phone access?
- Which information is stored in the "knowledge base" you are talking about?
- What do the authors mean by the term "snd-to-end system"?

I suggest the author's solution could be further influenced by existing emergency solutions. Thus, look on the drafts of the IETF working group ecrit. They are looking for standards in such research fields.

I have read this submission. I believe that I have an appropriate level of expertise to confirm that it is of an acceptable scientific standard.
